# Translating novel findings of perceptual-motor codes into the neuro-rehabilitation of movement disorders

**DOI:** 10.3389/fnbeh.2015.00222

**Published:** 2015-08-21

**Authors:** Mariella Pazzaglia, Giulia Galli

**Affiliations:** ^1^Dipartimento di Psicologia, Università degli Studi di Roma “La Sapienza”Rome, Italy; ^2^IRCCS Santa Lucia FoundationRome, Italy

**Keywords:** action observation, plasticity, rehabilitation, brain stimulation, motor cortex, multisensory rehabilitation

## Abstract

The bidirectional flow of perceptual and motor information has recently proven useful as rehabilitative tool for re-building motor memories. We analyzed how the visual-motor approach has been successfully applied in neurorehabilitation, leading to surprisingly rapid and effective improvements in action execution. We proposed that the contribution of multiple sensory channels during treatment enables individuals to predict and optimize motor behavior, having a greater effect than visual input alone. We explored how the state-of-the-art neuroscience techniques show direct evidence that employment of visual-motor approach leads to increased motor cortex excitability and synaptic and cortical map plasticity. This super-additive response to multimodal stimulation may maximize neural plasticity, potentiating the effect of conventional treatment, and will be a valuable approach when it comes to advances in innovative methodologies.

## Introduction

The inextricable link between action perception and execution was first posited by ideomotor theory and neurophysiological studies on mirror neurons (Prinz, 1990; di Pellegrino et al., 1992; Hommel, 1996). Within the past few years, it has been accepted that the bidirectional flow of perceptual and motor information can be useful in neurorehabilitation (Franceschini et al., 2012; Kantak and Winstein, 2012; Buccino, 2014). More specifically, theory-based evidence (Pomeroy et al., 2005; Garrison et al., 2010) has suggested that combined perceptual-motor training is beneficial in recovering and restoring motor ability after stroke (Small et al., 2012, 2013). Recently, this approach has been successfully applied to a considerable number of experimental lines of research validating how action observation is an effective way to enhance the performance of a specific motor skill (for a review, see Buccino, 2014).

## Action Observation Treatment

### Observation/Motor Training

During the rehabilitation protocol, patients are typically required to observe a specific movement that is presented in a video clip or demonstrated by an examiner, and contemporaneously (or thereafter) execute what they observe. A match or mismatch between visual signals and one’s own motor output re-informs the brain about how the limbs or mouth, for example, should move in order to successfully execute a gesture when a motor command is sent. Accurately reproducing spatial (Heyes and Foster, 2002), temporal (Badets et al., 2006a,b), and inter-limb coordination (Buchanan and Dean, 2010) characterizes movements, facilitates the generation of errorless motor patterns, and/or stimulates online correction output (Hecht et al., 2001; Heyes and Foster, 2002; Casile and Giese, 2006).

Behavioral and neurophysiological studies using combined visual-motor programs have suggested that the observation of a movement can improve motor performance in patients who have suffered a chronic ischemic stroke (Ertelt et al., 2007, 2012; Franceschini et al., 2010, 2012; Ertelt and Binkofski, 2012; Sale and Franceschini, 2012; Bang et al., 2013; Bonifazi et al., 2013; Brunner et al., 2014; Kim et al., 2014b; Marangon et al., 2014; Park et al., 2014a; Sale et al., 2014), patients with Parkinson’s disease (PD; Pelosin et al., 2010, 2013; Buccino et al., 2011; Esculier et al., 2014), and in children with cerebral palsy (Sgandurra et al., 2011, 2013; Buccino et al., 2012; Kim and Lee, 2013; Kim et al., 2014a). In the absence of brain injury, this type of treatment can be easily used to benefit patients with poor motor function or decline due to aging (Celnik et al., 2006; Bellelli et al., 2010; Park et al., 2014a). For example, bidirectional perceptual/motor training can be effective in patients with musculoskeletal injuries, such as those undergoing orthopedic surgery for the hip or knee (Bellelli et al., 2010; Park et al., 2014a,b), or in elderly individuals with reduced cognitive ability (Celnik et al., 2006; for a summary of these studies please see Table 1).

**Table 1:** **Summary of action observation treatment studies**.

Patology	Number of participants		Sessions	
	Experimental group	Control group	Number	Duration (minutes)	Type of action	Control	Training	Re-test (days)	Generalization	Reference
**Stroke**	33	34	40	15	Upper limbs daily actions	Static images of objects	Imitation	120 − 150	+	Sale et al. (2014)
	28	0	20	40	Daily actions with objects		Imitation	60	+	Franceschini et al. (2010)
	11	10	12	30	Functional walking tasks	Landscape images	Imitation		+	Park et al. (2014a)
	15	15	20	40	Treadmill walking actions	Nature video	Physical + Imitation			Bang et al. (2013)
	9	9 + 9	20	30	Dynamic balance + Gait abilities	Motor imagery + Physical training	Physical + Imitation			Kim and Lee (2013)
	8	8	18	90	Daily life hand and arm actions	Geometric symbols and letters	Imitation	56	+	Ertelt et al. (2007)—fMRI
**Parkinson’s disease**	8	8	18	40	Wii Fit game avatar actions	Rest + Motor imagery + Imitation			+	Esculier et al. (2014)—TMS
	7	8	NA	NA	Daily actions	Video clips with no motor content	Imitation		+	Buccino et al. (2011)
	10	10	12	60	Walking actions + Gait abilities	Landscape images	Imitation	28	+	Pelosin et al. (2010)
	10 + 10	14 + 8 + 10	1	6	Repetitive finger movements	Acoustic cue + Static hand	Imitation	2		Pelosin et al. (2013)
**Cerebral palsy**	12	12	15	60	Upper limbs daily actions	Computer games	Imitation	7 − 56 − 168	+	Sgandurra et al. (2013)
	8	7	15		Upper limbs daily actions	Video clips with no motor content	Imitation		+	Buccino et al. (2012)
	8	8	12	30	Upper limbs daily actions	Landscape images	Physical + Imitation	14	+	Kim et al. (2014a)
**Orthopedic**	30	30	18	24	Whole body daily actions	Video clips with no motor content	Imitation	7 − 14 − 21	+	Bellelli et al. (2010)
	9	9	9	40	Whole body daily actions	Physical training	Imitation		+	Park et al. (2014b)

Even in the brain of motor skill experts such as athletes or musicians, simultaneous training on the execution of a motor act during observation of an action results in better motor outcome (Haslinger et al., 2005; Mann et al., 2010).

### Positive Impact of Action Observation/Execution Treatment

Rehabilitative treatments based on perceptual-motor codes produce more effective results than motor acts that are mentally simulated (Gatti et al., 2013), motor training alone, or action observation alone (Hecht et al., 2001; Casile and Giese, 2006). Further, this rehabilitative approach seems to work quicker, and is more effective and more stable over long duration (Ertelt et al., 2007). One of the most striking advantages of this treatment is that it does not target one specific region of the body. In other words, this approach can be used to guide any biological effector (mouth, upper limbs, lower limbs, and trunk) in the production of an action.

During early stages of illness, motor observation and imagery could prevent the cortico-motor depression that is caused by limited use of the limbs (Bassolino et al., 2014). In the sub-acute phase, these two factors could stimulate and enhance the beneficial effects of motor training (Sale et al., 2014).

Several studies have recently reported the positive effects of motor imagery training on balance and gait performance (Dunsky et al., 2008), as well as on upper-limb function (Page et al., 2001), in patients with chronic stroke (Ietswaart et al., 2011) and on lower-limb function following spinal cord injury (Hotz-Boendermaker et al., 2011). Thus, it has been suggested that action observation could be a complementary training to facilitate the effect of an imagined motor task.

However, as observation of a movement provides unequivocal visual stimuli to the observer, it facilitates actual motor output more than imagining motions *per se*. The precise execution dynamic of an observed motor act could inform the motor imagery to improve the training supporting the kinesthetic aspects of the action, both when the classic motor training is not yet possible and after during the combined perceptual-motor rehabilitation treatments.

Despite the fact that training is enhanced when it is simultaneously combined with both action observation and execution (Stefan et al., 2005; Celnik et al., 2006, 2008), or when observation follows physical practice during early consolidation, some issues remain and further improvements can be made. For example, the time course of motor consolidation should be investigated in order to determine if it occurs flexibly across multiple timescales. Moreover, it is unclear whether action observation training is effective due to the recall of physical practice or motor performance *per se*, or because motor memories are relearned.

## Towards Multimodal Prediction in Rehabilitation

### The Effect of Multimodal Experience on Perceptual-Motor Codes

It is clear that the motor memory of an action is a multimodal experience that is modulated by visual (Haslinger et al., 2005), auditory (Kaplan and Iacoboni, 2007), and even olfactory (Pazzaglia, 2015) input. Given the mechanism that unifies action perception and action execution, it is highly plausible that, in rehabilitation, the consolidation of an early motor memory or recollection of a motor gesture may benefit from the use of different perceptual cues during the practice of physical actions. That is, perceptual cues might serve to recall motor-memory traces previously associated with a natural multisensory environment that cannot be retrieved from actual gestural knowledge. We know that effective approaches in rehabilitation are often intensive and repetitive, and therefore multisensory stimulation may facilitate the maintenance of attention and motivation in people undergoing therapy.

Studies in patients with brain damage demonstrate that both visual transitive and intransitive actions (Pazzaglia et al., 2008b), as well as hand and mouth action-related sounds, can be impaired (Pazzaglia et al., 2008a) if intentionally executed (Pazzaglia, 2013; Pazzaglia and Galli, 2014). One approach that has proven more powerful than vision alone in producing stabilized movements is using combined motor and auditory stimuli to encourage regularity of motor coordination in patients’ groups (Semjen and Ivry, 2001; Thaut et al., 2002). Additionally, in the olfactory domain, individuals with autism in the presence of a facilitating olfactory cue are able to successfully initiate imitation behavior (Parma et al., 2014). Interestingly, the mere perception of breast odors in infants induces immediate motor responses, such as directional crawling and sucking behavior (Varendi and Porter, 2001). Moreover, an olfactory visuomotor priming paradigm can induce facilitation effects regarding the time taken to process movement, favoring less severe bradykinesia and hand movement hypometria in patients with PD (Parma et al., 2013). Therefore, it is reasonable to think that emphasizing auditory, olfactory, and somatic perception, rather than exclusively focusing on the learning of simple visual-motor skills, may be potentially useful for relearning goal-directed actions.

A multimodal approach can even be employed in simple gestures, such as eating (or re-learning to eat) an apple, which is characterized by a variety of sensory perceptions (the color of the apple, the crunchy sound of its bite, the position of the fingers to grasp the apple, and the apple’s smell and taste). In this case, the motor re-education of a crucial daily life ability can be positively influenced by boosting multiple sensory modalities.

Importantly, augmenting stimulation by the combination of different modalities (olfaction, hearing, and haptics by vibrotactile actuators) may allow patients to recover lost motor functions as quickly and as permanently as possible. Indeed, multimodal integration may augment perceptual accuracy and saliency by providing redundant cues or by sustaining the missing information in perception reconstruction (Lenggenhager et al., 2013). Therefore, augmented multimodal training can reveal benefits not only in terms of outer signals, such as perceptual motor functions, but also via other neurorehabilitation interventions on inner signals involving training of motor tasks, as documented in experimental (Tsakiris et al., 2011; Ainley et al., 2014) and clinical conditions (Villiger et al., 2013; Lucci and Pazzaglia, 2015). Unfortunately, studies on the role of additive multimodal stimulation in triggering action representations during rehabilitation are currently lacking.

Although perceptual and motor event coding is crucial for shaping and implementing motor plans, knowledge about the predicted sensory and motor effects of one’s movement may provide further information that is useful for controlling and adjusting representations of an intended action.

### The Effect of Anticipatory Coding on Perceptual-Motor Codes

A high level of motor performance requires good ability to predict the outcomes of a motor action, which is a function that the motor system is well designed to fulfill. Important theoretical models conceptualize that the human motor system is equipped with specific, rapid, and automatic mechanisms that are crucial to the prediction of external sensory signals (Schubotz, 2007) and forthcoming motor acts, thus functioning as an anticipatory device (Wolpert and Flanagan, 2001). Studies on perceptual-motor synchronization demonstrate that action control is not sensitive to a match between sensory input and motor output, rather, it is sensitive to synchrony between the perceived sensory and motor effects of one’s action, supporting the existence of an internal system that is independent of motor implementation (Sergent et al., 1993). For example, when participants are asked to tap their finger in synchrony with a periodic sequence of tones, their performance depends entirely on perceived representation. In other words, motor events are controlled by the anticipation of their effects (Repp and Penel, 2002). Clinical evidence on the prediction notion of the motor brain comes from studies conducted on patients with brain damage that suffer from action execution disorders (Fontana et al., 2012) in which there is no Readiness-Potential (RP), an electrophysiological marker of motor preparation (Schurger et al., 2012), in the spontaneous activity of their motor system. RP seems to result from forward model predictions of the motor system that automatically precede a self-initiated movement (Kilner et al., 2004). Within this context, the lack of RP exhibited by patients indicates that the inability to predict consequences of one’s own motor action is directly associated with a distorted motor implementation. Efforts have been directed at bridging the discontinuities between prediction and implementation of motor actions (Wolpert and Flanagan, 2001; Iacoboni, 2003), in order to increase the anticipation of error recognition and interaction with the external world. This account of the motor system ensures that a prediction can be generalized from actions (Kilner et al., 2004) to events (Schubotz, 2007), and might benefit from multisensory stimulation that draws on the sensorimotor system.

Indeed, anticipation is also stimulated by hearing and olfaction. Memorization of the temporal association between the perception of a sound and a movement that accompanies it is a key element of learning (Aglioti and Pazzaglia, 2011). Regarding olfaction, however, odor may force us to prepare for action, adjusting the variability that is attributable to motor implementation. Such a phenomenon is documented when we smell an odor and subsequently grasp for food (Rossi et al., 2008). These are attractive examples of multi-modal prediction facilitation; however, whether and how behavioral paradigms that have been *ad hoc* devised on the basis of predictive coding algorithms will be combined with state-of-the-art neurophysiological techniques is a topic that will need to be addressed in the future.

## Neural Underpinnings of Perceived and Executed Actions

Action observation treatment was inspired by studies of macaque mirror neurons, where a particular class of multimodal cells were observed to be active during action execution and action perception (di Pellegrino et al., 1992; Keysers et al., 2003). Owing to the invasiveness of the technique used to record neuron activity *in vivo*, direct evidence of double-duty visuo-motor units in humans has only been reported in one study (Mukamel et al., 2010). Neurons that share bidirectional “seeing and doing” information are located in the medial section of the frontal lobe and in the temporal cortices (Mukamel et al., 2010). However, based on the pooled responses of very large populations of neurons, non-invasive neuroimaging and transcranial magnetic stimulation (TMS) suggest a similar system of motor simulation during action observation (Fadiga et al., 1995; Buccino et al., 2001; Aziz-Zadeh et al., 2002; Gazzola and Keysers, 2009). Neural subpopulations code either perceived or executed actions that may be linked to the striking mirror property in the premotor cortex, supplementary motor area (SMA), inferior parietal lobule, cingulate gyrus, and cerebellum (Molenberghs et al., 2012).

Different cognitive neuroscience techniques and experimental protocols in healthy subjects and patients with brain damage have provided convergent evidence for the existence of a fronto-temporal-parietal network involved in a variety of sensory signals that trigger or modulate an action (Aglioti and Pazzaglia, 2010). For example, sound-into-action translation processes have been identified in the left dorsal and premotor cortices and inferior parietal lobe (Gazzola et al., 2006). Moreover, the merging of visual and auditory information enables individuals to anticipate and optimize their perceptual and motor behavior recruiting the SMA, premotor cortex and cerebellum (Chen et al., 2008). Causative information on the auditory mapping of actions has been provided by our study on patients with apraxia, where we identified a clear association between deficits in performing hand- or mouth-related actions and the ability to recognize the associated sound in the frontal cortex and parietal lobe in the left hemisphere (Pazzaglia et al., 2008a).

Even human odors communicate dynamic information about motor states (Pazzaglia, 2015). For example, a combination of olfactory and visual inputs facilitates the selection of goal-directed movements (Castiello et al., 2006), and odorant objects (for example grasping a smelled strawberry) can potentially activate the frontoparietal brain network in response to the sight of similar actions (Tubaldi et al., 2011), thus hinting at the crossmodal nature of action simulation. This bi-directional message passing in the motor system can be seen when an individual grasps for a smelled object (Rossi et al., 2008), which clearly indicates predictive coding (Tubaldi et al., 2011).

Therefore, the perception/execution system, even when apparently driven by one modality, may be largely modulated by multimodality (Pazzaglia, 2015).

## Maximizing Perceptual Motor Plasticity in Rehabilitation

### Neuroplastic Brain Potentialities of Observed and Executed Actions

Action-observation training promotes neural reorganization via an adaptive plasticity, which leads to behavioral success in motor performance (Wenderoth, 2015). The cortical origin of the plastic modification induced by the matching of an observed and performed action, is well illustrated by studies on system motor experts. More specifically, these studies have demonstrated that the motor repertoire “resonates” with that of observed, ongoing movements in the frontoparietal structures (Haslinger et al., 2005; Calvo-Merino et al., 2006). Moreover, after a stroke, neural changes in the motor area that are caused by observation interventions suggest a functional reorganization comparable to those evoked in the brains of expert motors. For example, TMS studies of healthy individuals and patients with brain damage (Stefan et al., 2005; Celnik et al., 2006, 2008) have provided direct evidence of increased motor cortex and cortico-spinal excitability as a result of the enhanced synaptic efficiency that reflects long-term potentiation (Rosenkranz et al., 2007). These TMS studies indicate that action observation drives reorganization in the primary motor cortex to strengthen the motor memory of an observed action in young (Stefan et al., 2005; mean age, 34 years) and elderly (Celnik et al., 2006; mean age, 65 years) subjects, as well as in patients with chronic brain damage (Celnik et al., 2008). It has also been shown that 4 weeks of active, 18-day-cycle visual/motor training significantly enhances motor function, with a significant rise in the activity of specific motor areas that possess mirror properties in patients with stroke (Ertelt et al., 2007). Conversely, massed, high-frequency rehabilitative training (300–1000 daily repetitions) based on execution alone elicits only minimal neural reorganization (Kleim et al., 2004). Further, the action-observation of grasping movements of either the right or left hand, results in increased cortico-spinal excitability when TMS activates muscles of the unaffected hand (Ertelt et al., 2007). Lateralized M1 hyperexcitability could promote plastic changes in excitatory/inhibitory circuits through cortico-cortical connections. Indeed, although selective hemispheric improvement of healthy brains undergoing motor training coupled with action observation suggests a major role of cortical activity in the left hemisphere (Hamzei et al., 2012), action observation and execution tend to be salient in both hemispheres (Gazzola et al., 2007). It is thus not surprising that after therapy, a significant increase in activity was observed in the bilateral ventral premotor cortex, bilateral superior temporal gyrus, SMA, and contralateral supramarginal gyrus during free object manipulation in a functional magnetic resonance imaging study in stroke patients (Ertelt et al., 2007). Even in a disrupted network, enhancement of motor activity during spontaneous gestures has been observed after therapy aimed to promote adaptive neuroplasticity to enhance motor recovery (Garrison et al., 2013). Thus, the neural structures underpinning action execution observation of both hemispheres are also expected to play a role in motor recovery. Rather than employ training of a specific action observation in favor of a nonparetic limb, clinicians may point to regions activated in response to specific action observation in favor of both paretic and nonparetic limbs (Garrison et al., 2013). This supports the idea that interconnected regions of the action network can be balanced in an inhibitory manner. It is important to note that focal brain ischemia induces profound synaptic rearrangement, even in neurons adjacent to the insulted region. When the affected hemisphere undergoes long lasting increases in excitability (Manganotti et al., 2002), it influences glutamatergic synapses leading to reorganization of the peri-infarcted area (Cárdenas-Morales et al., 2010). It is also possible that the sensorimotor cortex of the affected hemisphere, through a mechanism of locally reduced transcallosal inhibition, changes the cortico-cortical excitability of the intact contralateral sensorimotor cortex. Thus, changes in inter-regional cortical excitability of a network related to a specific training program can be balanced by long-term potentiation- and depression-like processes, as well as to inhibitory mechanisms modulated by GABAergic activity stimulating a process of homeostatic metaplasticity (Ridding and Ziemann, 2010). Moreover, short-term plastic changes induced by low frequency TMS in the motor stimulation circuit can also be useful in adults with moderate to severe traumatic brain injury (Nielson et al., 2015), and in focal hand dystonia (Kimberley et al., 2015). Therefore, combined motor perception is a powerful mechanism to generalize action recovery (see Table 1), even if it is not related to the observed and executed stimuli used during video-therapy (Ertelt et al., 2007).

Assuming that map plasticity and motor process are interrelated, as they appear to be, then the change in excitability could inhibit or facilitate neural mechanisms underlying action execution and open up new possibilities for complementary multisensory processing with cumulative effects (Blankenburg et al., 2008).

### Neuroplastic Brain Potentialities of Multimodal Actions

A prompt comparison at the cortical level between two sensorimotor representations of a movement—that which is “perceived” and that which is “performed”– is necessary to induce major plastic changes. The functional contribution of perceptual information could be extended to other modalities depending on motor connections between sensory networks. Accordingly, although unimodal input may trigger action representation, congruent multimodal input is more appropriate to provide an enriched sensory representation, which, ultimately, enables full-blown characterization of an action simulation. Moreover, different inputs could converge at the synaptic level of up-stream motor areas. For example, the pooled response of very large populations of visuo-audio-motor neurons may result in a super-additive effect; that is, stronger than the sum of the unimodal effect (Keysers et al., 2003; Kaplan and Iacoboni, 2007). In addition, the multimodal response on pooled responses of visuo-olfacto-motor populations of neurons induces further increase in activity of the simulation map.

We also suggest that the functional gain derived from multimodal integration may have origins not only in the cortex, but also induces parallel changes in spinal excitability. For example, both visual and tactile data (e.g., peripheral nerve input) lead to an increase in M1 excitability, thus inducing plasticity and encouraging the use of multimodal stimulation in clinical settings (Bisio et al., 2014). Indeed, it has been shown that 14 min of median nerve stimulation during an observed congruent movement significantly increases corticomotor excitability in the motor cortex and reduces GABAergic inhibition (Rosenkranz and Rothwell, 2003). Such changes, suggest that the re-afferent somatosensory feedback of the median nerve could generate super-additive and cumulative effects. Similarly, a clear increase in corticospinal motor facilitation during the observation of the grasping of unseen but smelt objects (Rossi et al., 2008) or during passive listening to sounds associated with bimanual actions (Aziz-Zadeh et al., 2004), has also been observed. However, neither peripheral nerve stimulation or action observation alone could induce a comparable effect. Thus, combining the re-afferent visual and somatosensory feedback of an action leads to an increase in the synaptic efficiency evoked by action execution in motor areas.

## Concluding Remarks

While research on the relationship between observed and executed actions in the context of stroke treatment and rehabilitation has a short history, it has already provided new insights into the complexity of the underlying neural mechanisms of visual-motor training. The observation of actions through a process of visual retrieval and selection results in the encoding of a representation of the most probable action, providing a powerful tool for overcoming intentional motor-gestural difficulties. Moreover, tailored interventions based on an individual’s ability to acquire new (or relearn old) motor-memory traces through multimodal and predictive models may be the most promising approach for the development of treatments for goal-directed action disorders.

It is clear that progress in this area, which has both theoretical and practical implications for the care of patients, requires functional and anatomical information to guide the application of rehabilitation procedures. The importance of the interplay of multiple factors, such as lesion size, lesion location, and elapsed time after stroke onset should be taken into account. It is also likely that in cases different from stroke, such as PD, cerebral palsy, and hemiparesis, these factors interact with many more unidentified elements such as age at diagnosis, disease subtype, cognitive status, and baseline motor functions. Thus, targeting rehabilitation approaches on the basis of specific brain structures that mediate the effects of latent plasticity of complex interacting networks to facilitate recovery of function is an important challenge in this growing clinical field.

## Funding

MP received research support from “Sapienza” University of Rome, Grant FARI, 2011 – prot. C26I12TE94.

## Conflict of Interest Statement

The authors declare that the research was conducted in the absence of any commercial or financial relationships that could be construed as a potential conflict of interest.
